# A Placenta Previa and Double Births

**Published:** 1870-10

**Authors:** 


A Placenta Previa and Double Births is recorded by Dr. Maquida, of Mississippi, in which the mother and both children were saved. The first child presented by the breech, and crowded aside the placenta without wholly detaching it from the uterine walls; it was a small child. The second presented by the occiput, forcing down the placenta and directly occluding the os. With a little assistance the placenta was wholly detached from the uterine walls, and the delivery completed with no excessive hemorrhage.—*N. O. Med. Jour.*

